# Genetic Variations and Clinical Features of *NPHS1*-Related Nephrotic Syndrome in Chinese Children: A Multicenter, Retrospective Study

**DOI:** 10.3389/fmed.2021.771227

**Published:** 2021-11-11

**Authors:** Liping Rong, Lizhi Chen, Jia Rao, Qian Shen, Guomin Li, Jialu Liu, Jianhua Mao, Chunyue Feng, Xiaowen Wang, Si Wang, Xinyu Kuang, Wenyan Huang, Qingshan Ma, Xiaorong Liu, Chen Ling, Rong Fu, Xiaojie Gao, Guixia Ding, Huandan Yang, Mei Han, Zhimin Huang, Qian Li, Qiuye Zhang, Yi Lin, Xiaoyun Jiang, Hong Xu

**Affiliations:** ^1^Department of Pediatrics, The First Affiliated Hospital, Sun Yat-sen University, Guangzhou, China; ^2^Department of Nephrology, Children's Hospital of Fudan University, Shanghai, China; ^3^Department of Nephrology, The Children's Hospital, Zhejiang University School of Medicine, Hangzhou, China; ^4^Department of Nephrology and Rheumatology, Wuhan Children's Hospital (Wuhan Maternal and Child Healthcare Hospital), Tongji Medical College, Huazhong University of Science & Technology, Wuhan, China; ^5^Department of Nephrology and Rheumatology, Children's Hospital of Shanghai Jiaotong University, Shanghai, China; ^6^Department of Pediatric Nephrology, First Hospital, Jilin University, Changchun, China; ^7^Department of Nephrology, Bejing Children's Hospital Affiliated to Capital University of Medical Science, Beijing, China; ^8^Department of Pediatrics, Puyang Oilfield General Hospital, Puyang, China; ^9^Department of Nephrology, Shenzhen Children's Hospital, Shenzhen, China; ^10^Department of Nephrology, Nanjing Children's Hospital Affiliated to Nanjing Medical University, Nanjing, China; ^11^Department of Nephrology, Xuzhou Children's Hospital, Xuzhou, China; ^12^Department of Nephrology, Children's Hospital of Dalian Medical University, Dalian, China; ^13^Department of Pediatrics, Affiliated Hospital of Guangdong Medical University, Zhanjiang, China; ^14^Department of Pediatric Nephrology, Rheumatism and Immunology, Shandong Provincial Hospital Affiliated to Shandong University, Shandong, China; ^15^Department of Pediatrics, Affiliated Hospital of Qingdao University, Qingdao, China

**Keywords:** *NPHS1*, variants, congenital nephrotic syndrome, children, multicenter, steroid resistance

## Abstract

**Introduction:** Few studies have addressed the genetic spectrum of *NPHS1* variants in Chinese children with nephrotic syndrome. In this multicenter study, the clinical manifestations and features of *NPHS1* variants in Chinese children with nephrotic syndrome were researched.

**Method:** Genotypical and phenotypical data from 30 children affected by *NPHS1* variants were collected from a multicenter registration system in China and analyzed retrospectively.

**Results:** The patients were divided into two groups: congenital nephrotic syndrome (CNS [*n* = 24]) and non-CNS (early onset nephrotic syndrome [*n* = 6]). Renal biopsy was performed on four patients in the non-CNS group, revealing minimal change disease in three and focal segmental glomerulosclerosis in one. A total of 61 *NPHS*1 variants were detected, involving 25 novel variants. The “recurrent variants” included c.928G>A(p.Asp310Asn) in eight patients with CNS, followed by c.616C>A(p.Pro206Thr) in four, and c.2207T>C (p.Val736Ala) in three. Steroid treatment was applied in 29.2% (7/24)of the patients in the CNS group and 50% (3/6) of the patients in the non-CNS group. One patient in each group experienced complete remission but relapsed subsequently. Immunosuppressants were administered to three patients in the non-CNS group, eliciting an effective response. In the CNS group, three patients underwent renal transplantation and six died mainly from infection.

**Conclusion:** Variants of *NPHS1* cause CNS and early childhood-onset nephrotic syndrome. *NPHS1* variants in Chinese individuals with nephrotic syndrome (NS) were mainly compound heterozygous variants, and c.928G>A(p.Asp310Asn) in exon 8 may act as a recurrent variant in the Chinese population, followed by c.616C>A(p.Pro206Thr) in exon 6. Steroids and immunosuppressants may be effective in selected patients.

## Introduction

Nephrotic syndrome (NS) is one of the most common glomerular diseases in children. It is generally divided into steroid-sensitive NS (SSNS) and steroid-resistant NS (SRNS), depending on the response of the patient to steroid therapy. SRNS is a challenging clinical disease, in which 50% of the patients progress to end-stage renal disease within 15 years (1, 2). However, in some patients, temporary or sustained remission may be achieved. Some patients exhibit multidrug-resistant phenotypes, even with enhanced immunosuppressive therapy (3). In children with SRNS, there are usually genetic mutations affecting either podocytes or the glomerular basement membrane (4). *NPHS1* is one of the most common genetic SRNS causes, accounting for 13% of the genetic cases (3). The human *NPHS1* gene is located on the long arm of chromosome 19 (19q13.1) and contains 29 exons, whose protein product “nephrin” is a member of the immunoglobulin-like superfamily. *NPHS1* mutations are primarily responsible for CNS of the Finnish type. SRNS caused by mutations in the *NPHS1* gene is manifested by NS but lacks its extrarenal manifestations, and virtually all patients are unresponsive to steroid and immunosuppressant therapy (5). Fortunately, the recurrence rate after transplantation is low. SRNS can be divided into three types according to age: congenital (presenting within the first 3 months of life, most of which are steroid-resistant), childhood, and adulthood (6). As the *NPHS1* variants are not common, it may be difficult to identify them before genetic testing has been carried out in cases with late-onset NS. Furthermore, pediatric clinicians have a relatively insufficient understanding of this condition. To better understand *NPHS1* variants in pediatric patients in China, we present the clinical and genetic data from a pediatric study involving 30 patients, derived from a multicenter registration system. In this research, the gene mutation spectrum and the resultant clinical manifestations in children were analyzed aiming to describe the general situation of *NPHS1* variants in children with NS in China and raise awareness of this disease among clinical pediatricians.

The National Multicenter Registry (Chinese Children Genetic Kidney Disease Database [CCGKDD], www.ccgkdd.com.cn) has assembled the largest genetically screened cohort with pediatric renal disease in China (7). The data was submitted to the CCGKDD registry monthly by the participating centers in the nation. Eligibility criteria for the registration were complete information of phenotype and genotype for each family, and each medical center identified all the eligible patients post standardized training from the “Internet Plus” Nephrology Alliance of National Center for Children's Care. The phenotype data was identified by clinician experts in pediatric nephrology. The data-entry clerks who had only the ID number for the probands were trained to collect the phenotype information for the registration and they checked the birthdate and phenotype for duplication, and finally contacted clinicians to confirm the individual information before the final registry version. We retrospectively collected information regarding the genotype and phenotype of *NPHS1*-associated kidney disease from the registry and investigated the associations between clinical and genetic findings.

## Materials and Methods

### Study Design and Participants

Patients from 0 to 18 years of age diagnosed as NS combined with confirmed *NPHS1* variants, who underwent genetic analysis between January 1, 2014, and December 31, 2020, were retrospectively recruited from the CCGKDD in this cohort. NS was diagnosed according to the following criteria: heavy proteinuria (urine protein > 50 mg/kg/day); hypoalbuminemia (ALB <25 g/L); hypercholesteremia (cholesterol > 5.7 mmol/L); and clinical edema. All patients in this cohort were identified with *NPHS1* variants through genetic testing of the clinical panel (targeted gene sequencing) or whole exome sequence (WES). The information on presenting clinical features, genetic diagnosis, medical management, and status (with native renal function, dialysis, transplantation, deceased) at last follow-up was collected. The collection of data was stopped at 18 years of age. No identifying information was collected about the patients. A retrospective analysis of genotype, phenotype, and renal outcome was performed.

### Mutation Analysis

Due to the high cost of genetic testing for WES, targeted gene sequencing was more commonly used before 2017, whereas the application of WES gradually increased after 2018. Therefore, either the clinical panel (targeted genes sequencing) or WES was applied for genetic testing in an individual patient in this retrospective study. The 249 targeted genes included in the clinical panel (targeted gene sequencing) in this cohort are presented in Supplementary Table 1. WES and variant burden analysis were performed by Wuxi NextCODE, Chigene, and MyGenostics, respectively. Genomic DNA was isolated from blood lymphocytes and subjected to exome capture using Agilent SureSelect human exome capture arrays (V5, Life Technologies), NimbleGen, the xGen Exome Research Panel v1.0 (IDT), or MyGenosticsGencapTM capture technology, followed by next-generation sequencing on the Illumina HiSeq sequencing platform. Over 99% of the target sequence was sequenced at a 30 × read depth. Reads with adaptors, reads in which unknown bases (Ns) were more than 10%, and low-quality reads were discarded from raw data to generate clean reads. Clean reads were mapped to the human reference genome assembly (NCBI build 37/hg19) with CLC Genomics Workbench (version 6.5.1) software (CLC bio). Variants were annotated for predicted effects on protein function (using ANNOVAR and SnpEff) and allele frequency in public databases (genomAD, dbSNP, the 1000 Genomes Project, and ExAC). For synonymous variants, intronic variants that were more than 15 bp from exon boundaries (which are unlikely to affect messenger RNA splicing) and common variants (minor allele frequency >1%) were discarded. Missense variants were assessed with MutationTaster2, Provean, SIFT, and Polyphen-2. Evidence for disease causality was assessed using ClinVar and the Human Genome Mutation Database, followed by a manual review of the cited primary literature. Variant interpretations were performed by a panel of nephrologists or molecular geneticists with domain expertise in inherited kidney diseases, bioinformaticians, and clinical molecular geneticists using American College of Medical Genetics and Genomics (ACMG) guidelines (8) for clinical sequence interpretation. Diagnostic variants were defined as “pathogenic” or “likely pathogenic” or “variants of uncertain significance (VUS).” The novel variants of VUS in patients included in our study were considered as disease-causing based on the following: the phenotype of the patient or family history is highly specific for NPHS1-related NS; along with a pathogenic variant detected in trans; through clinical discussion combined with genotype and phenotype among nephrologists and molecular geneticists.

### Statistical Tests

Continuous variables summarized with median, interquartile range (IQR), and categorical data were summarized with proportions. Fisher's exact test or chi-square test was used to compare proportions depending on the number of cases. The level of significance was determined at *p* < 0.05. Statistical analysis was performed with SPSS version 20.0 statistical package software (IBM Co., Armonk, NY, USA).

### Ethics Statement

The present study adhered to the principles of the 1964 Declaration of Helsinki and was approved by the ethics committee of the participating centers. Written informed consent was obtained from the parents or guardians of all the patients for the publication of any potentially identifiable images or data included in this article. The Institutional Review Board (IRB) of the Children's Hospital of Fudan University (Shanghai, China) approved and monitored this study involving participating centers (IRB No. 2018286).

## Results

### Patient Characteristics and Clinical Phenotypes

In this study, data of 30 children with renal disease putatively caused by *NPHS1 variants* were collected from CCGKDD, which had included the data of 2,297 patients (30/2,297, counting for 1.30%). The median age at the time of onset was 51 days (range 1 day−3.6 years of age), the median age of genetic diagnosis was 2 months (1 month−3.6 years of age), the average duration at the time of genetic diagnosis was 1 month, and the male-to-female ratio was 1:1 (more details shown in Table 1). The patients were divided into two groups according to clinical phenotype:congenital NS (CNS[*n* = 24]); and non-CNS (early onset NS[*n* = 6]). There was no significant difference in the sex ratio between the CNS and the non-CNS groups. At the onset of the disease, nephrotic proteinuria and edema were the primary manifestations. Some were accompanied by microscopic hematuria (11/30 [36.7%]), oliguria (7/30 [23.3%]), and hypertension (4/30 [13.3%]). Regarding complications, some of the children suffered mild and moderate anemia (6/30 [20.0%]), congenital heart disease (3/30 [10.0%] including two with atrial septal defects and one with patent ductus arteriosus), congenital hypothyroidism (7/30 [23.3%]), and motor retardation (one case exhibiting an inability to crawl at 9 months). In terms of birth history, 12 premature infants, including five at the gestational age of 32–34 weeks, were associated with no amniotic fluid and placental abnormalities. Prematurity in one infant (32 weeks) was associated with hydramnios and a large placenta. Three patients had hernias (one indirect inguinal hernia and two umbilical hernias). One case was associated with cytomegalovirus infection. All the patients were examined for *NPHS1* variants. Renal biopsy was performed on four patients in the non-CNS group, among whom three exhibited minimal change disease (MCD) and one manifested with focal segmental glomerulosclerosis (FSGS). Serum albumin was lower in the CNS group than in the non-CNS group, and the urinary protein-to-creatinine ratio was higher in the CNS group. There was no significant difference in the levels of serum creatinine and cholesterol between the two groups (refer for more details in Table 2).

**Table 1:** Clinical features and treatment for 30 patients with *NPHS1* variants.

**ID**	**Age of onset/ gender**	**Renal histopathology**	**Steroid response**	**Treatment**	**Extrarenal manifestation**	**Outcome at the last follow-up**
**Congenital nephrotic syndrome**						
01	42d /M	nd	nd	Conservative treatment	Congenital hypothyroidism	CKD stage 1, NR
02	1mo/F	nd	PR	Steroid, renal replace treatment	Congenital clubfoot,hypertension, cholestasis	renal transplantation
03	2mo/M	nd	nd	Conservative treatment	None	CKD stage 1, NR
04	7d/M	nd	nd	No treatment	Congenital hypothyroidism	CKD stage 1, NR^*^
05	2mo/M	nd	nd	No treatment	Congenital heart disease (atrial septal defect)	Died of infection 5 months after birth
06	45d/M	nd	NR	Steroid	Hydrocele, umbilical hernia	CKD stage 1, NR
07	2mo/F	nd	nd	No treatment	Congenital hypothyroidism	CKD stage 1, NR^*^
08	1mo/F	nd	nd	Conservative treatment, renal transplantation	None	Renal transplantation
09	45d/F	nd	nd	No treatment	None	CKD stage 1, NR^*^
10	73d/F	nd	PR	Steroid	Inguinal hernia	CKD stage 1, NR^*^
11	3mo/M	nd	nd	No treatment	Congenital hypothyroidism	CKD stage 1, NR^*^
12	1mo/F	nd	CR	Steroid	None	CKD stage 1, CR
13	52d/F	nd	nd	No treatment	None	Died of infection after discharge
14	1mo/F	nd	nd	No treatment	Cystic lesion in the left frontal lobe	CKD stage 1, NR^*^
15	1mo/F	nd	nd	No treatment	None	CKD stage 1, NR^*^
16	2mo/F	nd	nd	Conservative treatment, ACEI	CMV infection; Loss of high-frequency hearing	CKD stage 1, NR
17	1mo/M	nd	nd	Conservative treatment, ACEI	Central hypothyroidism	Renal transplantation
18	1.5mo/M	nd	nd	Conservative treatment, ACEI	None	CKD stage 1, NR
19	1d/M	nd	nd	No treatment	PDA	Died of infection 15 days after birth
20	2d/F	nd	NR	Steroid	Femoral vein thrombosis	Died of infection 2 months after birth
21	1d/M	nd	nd	No treatment	None	Died of infection 11 days after birth
22	1d/F	nd	nd	Conservative treatment, ACEI	ASD	Died of infection 4 months after birth
23	3mo/F	nd	nd	Conservative treatment, ACEI	Ileus, umbilical hernia	CKD stage 1, NR
24	2mo/F	nd	NR	Steroid, ACEI	Umbilical hernia Congenital hypothyroidism	CKD stage 1, NR
**Early children onset nephrotic syndrome**						
25	8mo/M	FSGS	nd	Steroid, ACEI	None	CKD stage 1,PR
26	3.6y/M	MCD	nd	Steroid, ACEI	None	CKD stage 1, PR
27	2y/M	MCD	CR	Steroid, CsA	None	CKD stage 1, CR
28	1.5y/M	MCD	nd	ACEI	None	CKD stage 1, PR
29	1.5y/M	nd	NR	Steroid, tacrolimus, MMF	None	CKD stage 1, PR
30	3y/F	nd	NR	Steroid, CsA	None	CKD stage 1, PR

*nk, not known; MMF, mycophenolate mofetil; CsA, cyclosporin A; CR, complete remission; PR, partial remission; NR, no remission; CNS, congenital nephrotic syndrome; M, male; F, female; IS, immunosuppressant; FSGS, focal segmental glomerulosclerosis; MCD, minimal change disease; CKD, chronic kidney disease; nd, not done; CMV, cytomegalovirus; PDA, patent ductus arteriosus; ASD, atrial septal defect; NRDS, respiratory distress syndrome of newborn*.

* *The family gave up on treatment for the patient without follow-up since the first hospitalization*.

**Table 2:** The comparison between CNS group and non-CNS group in patients.

	**Total (*n* = 30)**	**CNS group (*n* = 24)**	**non-CNS group (*n* = 6)**
Patient No. (*n*, %)	100% (30/30)	80.0% (24/30)	20.0% (6/30)
Male. (*n*, %)	50.0% (15/30)	41.7% (10/24)	83.3% (5/6)
Median age at onset (day)	51 (30~82)	41 ± 26	771 ± 360
History of premature birth	40.0% (12/30)	50.0% (12/24)	0 (0/6)
Large placenta	13.3% (4/30)	16.7% (4/24)	0 (0/6)
**Laboratory result**			
Creatinine (umol/L)	20.0 ± 8.6	19.5 ± 9.0	21.7 ± 6.8
Albumin (g/L)	11.0 (10.0~15.6)	11.6 ± 3.4	24.2 ± 11.1
Cholesterol (mmol/L)	7.2 ± 4.0	6.8 ± 2.3	9.1 ± 8.3
UPCR (mg/mg)	24 (6.3~59.0)	54.4 ± 49.6	4.7 ± 2.4
Renal biopsy	13.3% (4/30)	0 (0/24)	66.7% (4/6)
**Treatment**			
Steroid treatment (*n*, %)	33.3% (10/30)	29.2% (7/24)	50.0% (3/6)
CR	20.0% (2/10)	14.3% (1/7)	33.3% (1/3)
PR	20.0% (2/10)	28.6% (2/7)	0 (0/3)
NR	60.0% (6/10)	57.1% (4/7)	66.7% (2/3)
Immunosuppressant (n, %)	10.0% (3/30)	0(0/24)	50.0% (3/6)
TAC+MMF (PR)	3.3% (1/30)	-	16.7% (1/6)
CsA (PR)	3.3% (1/30)	-	16.7% (1/6)
CsA (CR)	3.3% (1/30)	-	16.7% (1/6)
ACEI (n, %)	23.3% (7/30)	16.7% (4/24)	50.0% (3/6)
**Outcome**			
CKD stage 1	70.0% (21/30)	62.5% (15/24)	100% (6/6)
CKD stage 1, CR	6.7% (2/30)	4.2% (1/24)	16.7% (1/6)
CKD stage 1, PR	16.7% (5/30)	0 (0/24)	83.3% (5/6)
CKD stage 1, NR	46.7% (14/30)	58.3% (14/24)	0 (0/6)
Renal transplantation	10.0% (3/30)	12.5% (3/24)	0 (0/6)
Death	20.0% (6/30)	25.0% (6/24)	0 (0/6)
**Type of variant**			
Novel variants (n, %)	41.0% (25/61)	44.0% (22/50)	27.3% (3/11)
Missense (*n*, %)	49.2% (30/61)	46.0% (23/50)	63.6% (7/11)
Frameshift (*n*, %)	19.7% (12/61)	20.0% (10/50)	18.2% (2/11)
Splice (*n*, %)	11.5% (7/61)	14.0% (7/50)	0 (0/11)
Nonsense (*n*, %)	13.1% (8/61)	14.0% (7/50)	9.1% (1/11)
Intronic (*n*, %)	3.3% (2/61)	4.0% (2/50)	0 (0/11)
Duplication (*n*, %)	1.6% (1/61)	2.0% (1/50)	0 (0/11)
Nonframeshift deletion (*n*, %)	1.6% (1/61)	0 (0/50)	9.1% (1/11)
**Hotspot variants**			
c.928G>A	13.1% (8/61)	16.0% (8/50)	0 (0/11)
c.616C>A	6.6% (4/61)	2.0% (1/50)	27.2% (3/11)
c.2207T>C	4.9% (3/61)	4.0% (2/50)	9.1% (1/11)

### *NPHS1* Variants

**Figure 1:**
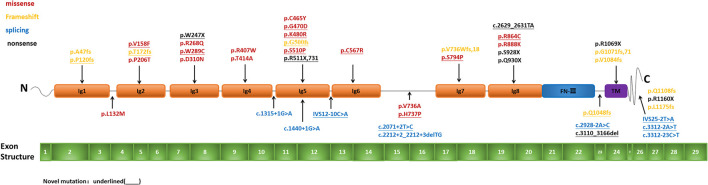
Pattern of variant(s) in the *NPHS1* gene: Exon structure of the *NPHS1* gene and the location of the variants identified in the present study.

**Figure 2:**
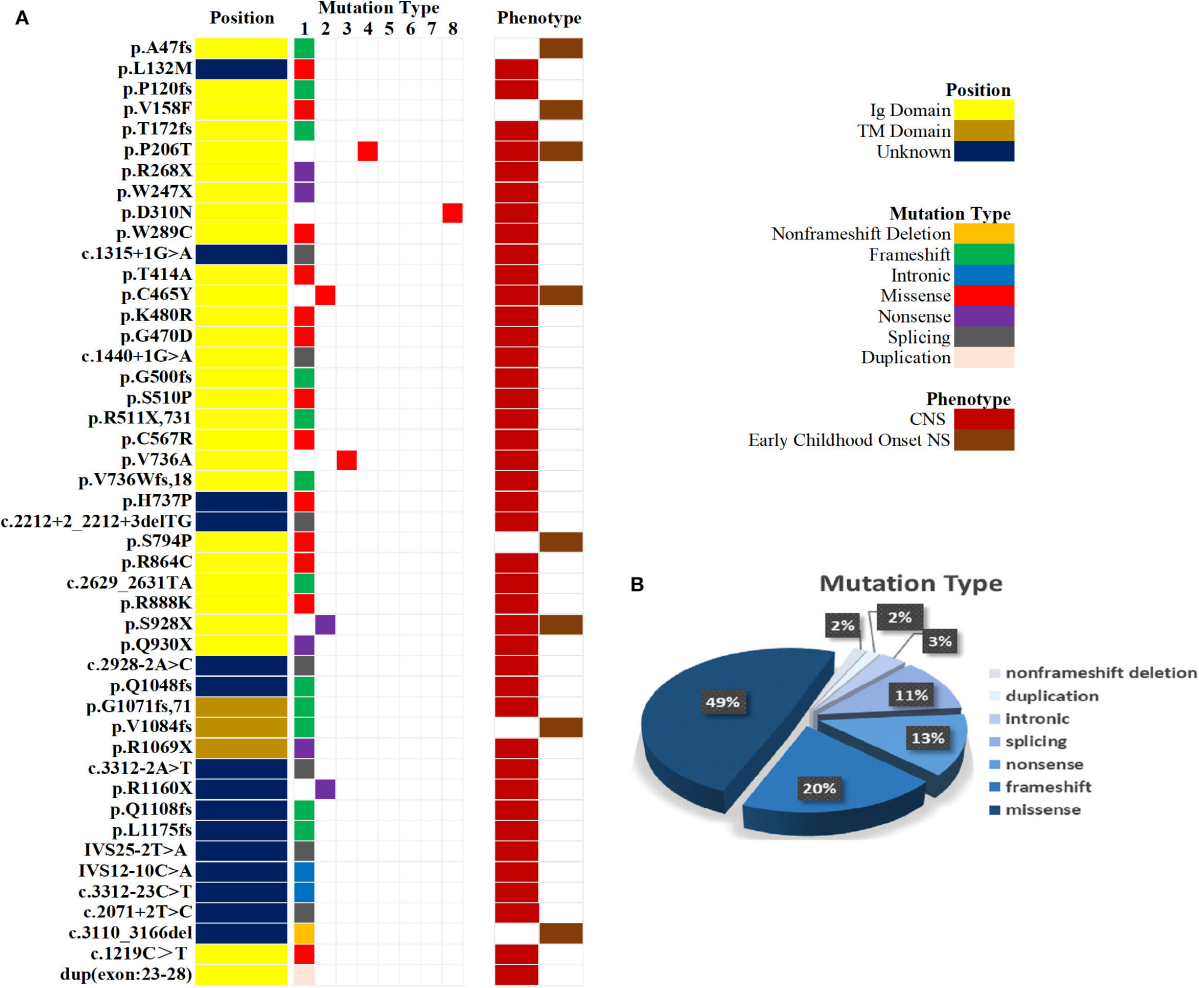
An overview of variants according to *NPHS1*gene type. **(A)** Mutation type and phenotype of *NPHS1* in 30 patients. **(B)** Percentages of variants according to *NPHS1* gene type.

Mutation analysis of all *NPHS1* genes was performed on 30 patients from 30 families. Nephrotic panel gene testing (targeted gene sequencing) was done in 20 patients and WES in the remaining 10 patients. Diagnostic variants including VUS combined with “likely pathogenic” (LP) or “pathogenic” (P) were thought to be disease-causing variants through assessment of the genotype and phenotype associations. The patients in our cohort were recruited with the variation of *NPHS1* gene, without the combination of any other genes variations. One patient exhibited a homozygous *NPHS1* variant (c.3110_3166del), and a single heterozygous *NPHS1* variant was identified in another, whereas the remaining 28 exhibited compound heterozygous *NPHS1* variants (more details in Table 3 and Supplementary Table 2). A total of 61 variants were detected among all the patients, including 46 different disease-causing *NPHS1* variants, with 25 novel variants that have not been previously reported, mainly in the CNS group. Mutation types consisted of 30 missense, 12 frameshifts, seven splice-site, eight nonsense, one duplication, and one deletion. The recurrent variant was c.928G>A in exon 8 in eight patients, followed by c.616C>A in four, c.2207T>C in three, and c.1394G>A, c.2783C>A, and c.3478C>T in two, respectively. The variants were broadly distributed over the nephrin protein located in exons 2, 3, 4, 6, 8, 10, 11, 13, 16, 18, 19, 20,22, 23, 24, 25, 26, 27, and 28 (Figure 1). The variant of c.928G>A was present in the CNS group, whereas c.616C>A was mainly found in the non-CNS group (more details in Table 2). The other variants exhibited no significant differences between the two groups. An overview of variants of NPHS1 gene type in this study is shown in Figure 2. The patients were classified into the P+P/LP+P group and P+VUS/LP+VUS group to review the clinical features of patients from the perspective of the features of the variants. The results of the comparison between the two groups with different disease-related pathogenicity are shown in Table 4. The comparison of the phenotype and genotype in CNS patients with *NPHS1* variants in this study with the other four cohorts reported in the literature (23–26) is shown in Table 5.

**Table 3:** Genetic information for 30 patients with *NPHS1* variants (NM_004646.3).

**Case**	**Age of onset/ gender**	**NHSP1 variants**			**Type of variant**	**Hom/Het**	**Variation origin**	**ACMG**	**Reference**	**MAF**	
		**Nucleotide change**	**Aminoacid change**	**Location**						**Gome_ALL**	**Gome_EAS**
**Congenital nephrotic syndrome**											
1	42d /M	IVS25-2T>A^*^	/	Intron 25	Splice site	Co-Het	Unknow	VUS	-	-	-
		c.928C>T	p.Asp310Asn	Exon 8	Missense	Co-Het	Unknow	LP	(9)	1.2156 ×10^−5^	1.634 ×10^−4^
2	1mo/F	c.3325C>T	p.Arg1109Ter	Exon 26	Nonsense	Co-Het	Unknow	P	(10)	1.551 ×10^−4^	1.0874 ×10^−4^
		c.3312-2A>T^*^	/	/	/	Co-Het	Unknow	VUS	-	-	-
3	2mo/M	c.2590C>T^*^	p.Arg864Cys	Exon 19	Missense	Co-Het	F	LP	-	4.8723 ×10^−5^	3.3025 ×10^−4^
		c.867G>T^*^	p.Trp289Cys	Exon 8	Missense	Co-Het	het; p,wt; m,wt	LP	-		
4	7d/M	c.1394G>A	p.Cys465Tyr	Exon 11	Missense	Co-Het	Unknow	P	(11)	-	-
		c.928G>A	p.Asp310Asn	Exon 8	Missense	Co-Het	F	P	(9)	1.2156 ×10^−5^	1.634 ×10^−4^
5	2mo/M	c.394G>A^*^	p.Glu117Lys	Exon 3	Missense	Co-Het	F	VUS	-	-	-
		c.1439A>G^*^	P.Lys480Thr	Exon 11	Missense	Co-Het	F	VUS	-	-	-
		c.1500_1507del^*^	p.Gly500fs	Exon 12	Frameshift	Co-Het	Not Available	P	-	-	-
6	45d/M	c.3478C>T	p.Arg1160Ter	Exon 27	Nonsense	Single Het	F	LP	(12)	9.943 ×10^−5^	5.4366 ×10^−5^
7	2mo/F	c.2629-c.2630delA AinsT^*^	p.Lys877Xfs^*^1	Exon 19	Frameshift	Co-Het	F	P	-	-	-
		c.1315 + 1G>A(-)	/	/	/	Co-Het	M	P	(13)	-	-
8	1mo/F	**c.2205_2206ins** TGGAC^*^	p.Val736Trpfs^*^18	Exon 16	Frameshift	Co-Het	M	P	-	-	-
		c.3478C>T	p.Arg1160Ter	Exon 27	Nonsense	Co-Het	F	LP	(12)	9.943 ×10^−5^	5.4366 ×10^−5^
9	45d/F	c.3213delG	p.Leu1072Phe fs^*^71	Exon 24	Frameshift	Co-Het	F	LP	(14)	-	-
		c.2663G >A	p.Arg888Thr	Exon 19	Missense	Co-Het	M	P	(15)	4.3654 ×10^−6^	0
10	73d/F	c.928G>A	p.Asp310Asn	Exon 8	Missense	Co-Het	M	P	(9)	1.2156 ×10^−5^	1.634 ×10^−4^
		c.360del C^*^	p.Pro120fs	Exon 8	Frameshift	Co-Het	F	P	-	-	-
		c.1240.A>G	p.Thr414Ala	Exon 8	Missense	Co-Het	F	VUS	Clinvar:VCV000930198	-	-
11	3mo/M	c.616C>A	p.Pro206Thr	Exon 6	Missense	Co-Het	M	LP	(16)	3.185 ×10^−5^	4.35 ×10^−4^
		IVS12-10C>A^*^	/	Intron 12	Splice site	Co-Het	F	VUS	-	-	-
12	1mo/F	c.928G>A	p.Asp310Asn	Exon 8	Missense	Co-Het	F	P	(9)	1.2156 ×10^−5^	1.634 ×10^−4^
		c.2207T>C	p.Val736Ala	Exon 16	Missense	Co-Het	M	P	(17)	-	-
		c.3312-23C>T	/	Intron 25	Missense	Co-Het	M	VUS	(18)	1.0608 ×10^−5^	1.5039 ×10^−4^
13	52d/F	**c.2928-2A>C(IVS21)** ^*^	/	Intron 21	Splice site	Co-Het	M	LP	-	-	-
		c.928G>A	p.Asp310Asn	Exon 8	Missense	Co-Het	F	P	(9)	1.2156 ×10^−5^	1.634 ×10^−4^
14	1mo/F	c.802C>T	p.Arg268X	Exon 7	Nonsense	Co-Het	F	LP	(19)	2.8512 ×10^−5^	0
		c.1528T>C^*^	p.Ser510Pro	Exon 12	Missense	Co-Het	M	VUS	-	-	-
15	1mo/F	c.2788C>T	p.Gln930X	Exon 20	Nonsense	Co-Het	F	P	(15)	-	-
		c.3442delC	p.Gln1148fs	Exon 27	Frameshift deletion	Co-Het	M	P	(15)	-	-
16	2mo/F	c.2207T>C	p.Val736Ala	Exon 16	Missense	Co-Het	M	P	(17)	-	-
		c.2210A>C^*^	p.His737Pro	Exon 16	Missense	Co-Het	F	VUS	-	-	-
17	1mo/M	c.2212 + 2_2212 + 3delTG	-	-	Splicing	Co-Het	F	P	(19)	-	-
		c.1409G>A^*^	p.Gly470Asp	Exon 11	Missense	Co-Het	M	VUS	-	-	-
18	1.5mo/M	c.1440 + 1G>A	-	Intron 11	Splicing	Co-Het	F	P	(20)	-	-
		c.928G>A	p.Asp310Asn	Exon 8	Missense	Co-Het	M	P	(9)	1.2156 ×10^−5^	1.634 ×10^−4^
19	1d/M	c.741G>A^*^	p.Trp247X	Exon 7	Nonsense	Co-Het	M	P	-	-	-
		c.928G>A	p.Asp310Asn	Exon 8	Missense	Co-Het	F	P	(9)	1.2156 ×10^−5^	1.634 ×10^−4^
20	2d/F	c.3144delG^*^	p.Gln1048fs	Exon 23	Frameshift deletion	Co-Het	M	P	-	-	-
		c.514delA^*^	p.Thr172fs	Exon 4	Frameshift deletion	Co-Het	F	P	-	-	-
21	1d/M	c.1699T>C^*^	p.Cys567Arg	Exon 13	Missense	Co-Het	F	VUS	-	-	-
		c.3523_3524del	p.Leu1175ValfsTer2	Exon 28	Frameshift deletion	Co-Het	M	LP	(21)	3.9765 ×10^−6^	5.4366 ×10^−5^
22	1d/F	c. 1531C > T^*^	p.Arg511X,731	Exon 12	Frameshift deletion	Co-Het	F	LP	-	-	-
		**c.2071** **+** 2T>C^*^	-	-	Splicing	Co-Het	M	P	-	-	-
23	3mo/F	c.2783C>A	p.Ser928X	Exon 20	Nonsense	Co-Het	Unknow	P	(22)	-	-
		c.928G>A	p.Asp310Asn	Exon 8	Missense	Co-Het	Unknow	P	(9)	1.2156 ×10^−5^	1.634 ×10^−4^
24	2mo/F	c.1219C>T	p. Arg407Trp	Exon 10	Missense	Co-Het	F	P	(23)	-	-
		dup(exon23-28)^*^	-	Exon 23-28	Duplication	Co-Het	M	LP	-	-	-
**Early children onset nephrotic syndrome**											
25	8mo/M	c.616C>A	p.Pro206Thr	Exon 6	Missense	Co-Het	M	LP	(16)	3.185 ×10^−5^	4.35 ×10^−4^
		c.472G>T^*^	p.Val158Fhe	Exon 4	Missense	Co-Het	F	VUS	-		
26	3.6y/M	c.2207T>C	p.Val736Ala	Exon 16	Missense	Co-Het	F	P	(17)	-	-
		c.616C>A	p.Pro206Thr	Exon 6	Missense	Co-Het	M	LP	(16)	3.185 ×10^−5^	4.35 ×10^−4^
27	2y/M	c.C2783A	p.Ser928X	Exon 20	Nonsense	Co-Het	M	P	(22)	-	-
		c.139delG	p.Ala47ProfsTer81	Exon 2	Frameshift	Co-Het	F	LP	(12)	8.1213 ×10^−6^	0
28	1.5y/M	c.3250dupG	p.Val1084fs	Exon 24	Frameshift insertion	Co-Het	F	LP	(10)	-	-
		c.2380T>C^*^	p.Ser794Pro	Exon 18	Missense	Co-Het	M	VUS	-	-	-
29	1.5y/M	c.1394G>A	p.Cys465Tyr	Exon 11	Missense	Co-Het	Unknow	P	(11)	-	-
		c.616C>A	p.Pro206Thr	Exon 6	Missense	Co-Het	Unknow	LP	(16)	3.185 ×10^−5^	4.35 ×10^−4^
30	3y/F	c.3110_3166del^*^	/	Exon 23	Nonframeshift deletion	Hom	hom; F, het; M, het	P	-	-	-

* *Novel variants in bold*.

*Hom, Homozygous; Het, Heterozygous; Co-Het, compound Heterozygous; MAF, Minor Allele Frequency; P, Pathogenic; LP, Likely Pathogenic; VUS, Variants of uncertain significance; m, month; y, year. D, day. Wt, wild type*.

**Table 4:** The comparison between two groups with different pathogenicity variants.

	**P+P/P+LPgroup *(n* = 19)**	**P+VUS/LP+VUSgroup *(n* = 11)**	***p* value**
Patient No. *(n*, %)	63.3% (19/30)	36.7% (11/30)	-
CNS *(n*, %)	78.9% (15/19)	81.8% (9/11)	0.380
Non-CNS *(n*, %)	21.1% (4/19)	18.2% (2/11)	0.739
Median age at onset(day)	57(32~88)	50(19~59)	0.400
History of premature birth	26.3% (5/19)	63.6% (7/11)	0.052
Renal biopsy	10.5% (2/19)	18.2% (2/11)	0.470
**Treatment**			
Steroid treatment *(n*, %)	42.1% (8/19)	18.2% (2/11)	0.210
CR	12.5% (1/8)	50% (1/2)	0.378
PR	25.5% (2/8)	0 (0/2)	-
NR	62.5% (5/8)	50% (1/2)	0.667
Immunosuppressant *(n*, %)	15.8% (3/19)	0 (0/11)	0.279
TAC+MMF (PR)	33.3% (1/3)		-
CsA (PR)	33.3% (1/3)		-
CsA (CR)	33.3% (1/3)		-
ACEI *(n*, %)	15.8% (3/19)	36.4% (4/11)	0.200
**Outcome**			
CKD stage 1	58.3% (14/19)	63.6% (7/11)	0.429
CKD stage 1, CR	14.3% (2/14)	14.3% (1/7)	0.726
CKD stage 1, PR	28.6% (4/14)	0 (0/7)	0.255
CKD stage 1, NR	57.1% (8/14)	85.7% (6/7)	0.127
Renal transplantation	5.3% (1/19)	18.2% (2/11)	0.298
Death	44.4% (4/19)	18.2% (2/11)	0.739
**Variant pathogenicity**	P/LP	VUS	
Variants *(n*, %)	78.7% (48/61)	21.3% (13/61)	-
**Mutation type**			
Novel mutation *(n*, %)	29.2% (14/48)	84.6% (11/13)	<0.001
Missense mutation *(n*, %)	41.7%(20/48)	76.9% (10/13)	0.025
Splice mutation *(n*, %)	8.3%(4/48)	23.1% (3/13)	0.159
Frameshift mutation *(n*, %)	25.0%(12/48)	0 (0/13)	0.054
Nonsense mutation *(n*, %)	16.7%(8/48)	0 (0/13)	0.183
Intronic mutation *(n*, %)	4.2%(2/48)	0 (0/13)	1.00
Duplication *(n*, %)	2.1% (1/48)	0 (0/13)	1.00
Nonframeshift deletion *(n*, %)	2.1% (1/48)	0 (0/13)	1.00

*P, Pathogenic; LP, Likely Pathogenic; VUS, Variants of uncertain significance; High pathogenicity: P/LP variants. Low pathogenicity, LP or P + VUS variants; CKD, chronic kidney disease*.

**Table 5:** Phenotype and genotype of CNS patients with *NPHS1* mutation in the literatures and comparison with our cohort.

		**CNS patients with** ***NPHS1*** **mutation in the Literature and in Our Cohort**				
		**Worldwide Cohort (23)**	**France (24)**	**Saudi Arabia (26)**	**Japan (25)**	**This study**
	Time interval	1996–2008	2000–2014	2008–2017	Nationwide survey in 2016	2014–2020
Phenotype	Patient number	36	36	9	33	30
	Male. *(n*, %)	NA	52.6% (19/36)	55.5% (5/9)	45.4%(15/33)	50.0% (15/30)
	CNS	100%	100%	88.9% (8/9)	100%	80.0% (24/30)
	Child onset NS *(n*, %)	0	0	11.1% (1/9,)	0	20.0% (6/30)
	Median age at onset(day)	NA	0.5 (0–13)	2.4 (0.1–6), month	0.0 (0.0–2.0),month	51.0 (30.0~82.0)
	History of family	NA	Consanguinity 46%	Consanguinity 100%	NA	0
	Large placenta	NA	12/17	-	30/30	13.3% (4/30)
	Extra-renal anomalies	NA	NA	Right pulmonary artery stenosis; Seizure disorder; Recurrent chest infection; Recurrent chest infection	Malformation; Epilepsy; Mental retardation	Congenital hypothyroidism, congenital clubfoot, hernia, CMV infection, Loss of hearing, ASD, PDA
	Renal biopsy	10/36	8/36	None	13/30	4/30
Treatment	Steroid treatment *(n*, %)	20.8%	None	NA	3/29 (nR, 3)	33.3% (10/30)
	IS *(n*, %)	4 in CsA(NR,4)	None	NA	3/28 (CSA, NR, 2; PR, 1)	10.0% (3/30)
Outcome	CKD stage 1	NA	NA	NA	NA	70.0% (21/30)
	RRT	10 in renal transplantation	68% (25/37)	2 with PD	26/33 in PD; 1/33 in HD; 17/33 in renal transplantation	10.0% (3/30) in renal transplantation
	Death	NA	16% (6/37)	70.1% death in CNS	NA	20.0% (6/30)
Mutation type	Homozygous *(n*, %)	26/36	61.1% (22/36)	100%	NA	3.3% (1/30)
	Co-het *(n*, %)	10/36	38.9% (14/36)	0	NA	96.7% (29/30)
	Novel mutation *(n*, %)	19/37	7/31	NA	NA	41.0% (25/61)
	Hotspot variants	c.1760T>G, 10.8% (4/37) c.3243_3250insG, 8.1% (3/37) c.3478C>T, 8.1% (3/37)	c.139 del; 13.9% (5/36) c.1379 G>A; 22.2% (8/36)	NA	NA	c.928G>A, 13.1% (8/61) c.616C>A, 6.6% (4/61) c.2207T>C, 4.9% (3/61)

*A, not applicable; CsA, cyclosporin A; CR, complete remission; PR, partial remission; NR, no remission; CNS, congenital nephrotic syndrome; RRT, renal replacement therapy; CKD, chronic kidney disease; PD, peritoneal dialysis; HD, hemodialysis; Co-het, Compound heterozygous; IS, Immunosuppressant*.

### Treatment and Follow-Up Information

All 24 patients with CNS received nutritional support, diuretics, albumin infusion, preventive measures, treatment of infection, and other symptomatic support treatment at disease onset. In total, 10 patients were administered steroid therapy, including three with early childhood-onset NS and seven with CNS. In the CNS group, three patients responded effectively, including two with partial remission and one with complete remission. In the non-CNS group, two patients were resistant to steroid therapy and one had complete remission but relapsed frequently. Immunosuppressive therapy, including cyclosporin A (CsA), tacrolimus, and mycophenolate, was administered to the three patients with childhood-onset NS, resulting in effective outcomes, including two with partial remission and one with complete remission. Seven patients were on antiproteinuric therapy with angiotensin-converting enzyme inhibitors, including four in the CNS group, exhibiting no response, and three in the non-CNS group, exhibiting partial response (more details provided in Table 2). Due to the poor socioeconomic status of the family or poor prognosis related to hereditary factors, 30.0% (9/30) of patients with CNS phenotypes were left untreated after genetic diagnosis. At the most recent observation, the remaining 70.0% (21/30) of patients were still in stage 1 chronic kidney disease (CKD) and 10.0% (3/30) had undergone renal transplantation. Among those who had received early preemptive kidney transplantation, one patient had undergone repeated transplantation due to allograft failure. All three patients had remained in stage 1 CKD before the first transplantation. Among the patients with stage 1 CKD (*n* = 21), two were in complete remission, five in partial remission, and 14 in no remission at the last observation.

## Discussion

The current study was a nationwide, multicenter study, with the largest number of pediatric cases of *NPHS1* gene variants in the country. This research yielded important information regarding the distribution of clinical phenotypes and genotypes in childhood SRNS. The 30 patients were from 30 unrelated families located in 11 provinces and autonomous regions in China. By analyzing the characteristics of clinical manifestations and genotypes, we aimed to enable clinicians to understand better disease-related *NPHS1* variants and optimize decision making.

In this study, most of the *NPHS1* variants were found in the CNS group, accounting for 80.0% (24/30) of cases. They were also found in early childhood-onset NS (age ≤ 3 years), accounting for 20.0% (6/30) of the cases. The results of the current study and previous findings confirm that *NPHS1* variants can cause a broader variety of clinical phenotypes in nephrotic syndrome including childhood- and adult-onset focal segmental glomerulosclerosis than CNS (3, 6, 23, 27, 28). The variants of NPHS1 showed no obvious difference between patients with MCD and FSGS. Similarly, the previous study showed the spectrum of renal histologic findings atypical for CNS with NHPS1 variants ranging from MCD to FSGS (6). The histopathological manifestations may be related to podocyte injury and persistent proteinuria caused by NPHS1 variants. Extrarenal manifestations were present in 16 of the 24 patients with CNS in our study, mainly in congenital hypothyroidism, hernia, and congenital heart disease. From the results, there was no considerable difference in variants between patients with and without extrarenal manifestations. Combined with the literature review, we consider that the extrarenal manifestations in our study were possibly secondary to CNS or cooccurring complications along with CNS rather than related to genetic variation.

A previous consensus indicated that patients with NS caused by *NPHS1 variants* exhibit a weak response to steroid therapy (5, 24, 29). In our study, one patient (case 12), responded effectively to initial steroid therapy and maintained an effective response in subsequent nonfrequent relapses. The *NPHS1* variants of this child exhibited three-compound heterozygous variants, namely c.928G>A in exon 8, c.2207T>C in exon 16, and c.3312-23C>T in intron 25. This finding from our study was inconsistent with previous reports and may indicate that steroid therapy can be usefully applied in CNS patients with these variants loci in *NPHS1*. However, it was just an individual observation result from clinical practice in our study. A recent genome-wide association study reported that there are some common risk variants in *NPHS1* that are associated with childhood SSNS (30). However, the steroid-sensitive mechanism remains unclear. We speculate that perhaps the nephrin protein, resulting from these variants, maintains its partial function and shows the response to steroid or immunosuppressive therapy, which may help actin reorganization in the cytoskeleton of podocytes. Further study of its molecular mechanism and long-term follow-up is required. In this study, it was also indicated that some children with early childhood-onset NS caused by *NPHS1* variants may be effectively treated with steroid and immunosuppressive agents. Studies involving greater numbers of patients will be needed to observe the effects of immunosuppressive therapy, along with further mechanism studies to determine the significance of these variants.

The results of *NPHS1* variant analysis in our study revealed that there were mainly compound heterozygous variants, accounting for 93.3% (28/30) of cases, with one homozygous variant. This finding suggests that compound heterozygous variants are the major variation pattern in *NPHS1* in Chinese patients. The most frequent variant was c.928G>A in exon 8 (eight patients), which was present in CNS patients, followed by c.616C>A in four, c.2207T>C in three, and c.1394G>A, c.2783C>A, and c.3478C>T in two, respectively. These findings were, to some extent, different from reports from other Chinese studies (15, 31–33). Our findings indicated that heterozygous variants, including c.928G>A and c.616C>A, are “pathogenic recurrent variants” of *NPHS1* in China, whereas c.928G>A is the main genotype of CNS. It is generally believed that recurrent genetic variants occur *via* two mechanisms: one is a founder effect and the other is a mutational hot spot. In our study, the variant 928G>A occurred with high frequency and was rarely reported in other populations, and so we speculated whether it is a founder effect. However, there remains the little specific supporting basis for this speculation since there is little data about researches on *NPHS1* in China, and data in our study was so limited that it was difficult to perform further analysis for the founder effect. The mutation results revealed that missense mutations were the main mutation types in the two groups. Splice mutations, nonsense mutations, and intronic mutations mainly occurred in patients with a phenotype of congenital nephrotic syndrome, similar to previous reports. In the present study, 13 variants were found to be of uncertain significance, accounting for 21.3% of cases, of which 11 variants have not been reported yet. Most of the patients who carried the novel variants, along with a pathogenic variant detected in trans, presented typical clinical features of CNS. Among the 11 novel variants of VUS, eight were located in exons with “damaging” *in silico* predictions. Although the pathogenicity of variants with VUS has not been identified, from the perspective of clinical practice based on factors involved, all of them in this study were thought to be disease-causing variants through fully integrated analyses of the phenotypical manifestations and genotypes. However, the introns and copies of these variants need to be further detected and analyzed to determine their pathogenicity.

In China, the prognosis for CNS was poor before the turn of the 21st century. Significant progress has been made in healthcare, as well as in pediatric nephrology during the past few decades, and the prognosis has improved significantly. In terms of treatment and prognosis for the 30 patients in this study, 12.5% (3/24) of children diagnosed with CNS received kidney transplantation. Up to 30.0% (9/30) of the patients did not receive appropriate treatment after genetic diagnosis because their parents declined treatment due to the high financial burden involved or concerns about poor prognosis. A cross-sectional nationwide survey of CNS and infantile NS in Japan reported that up to 78.8% (26/33) of patients underwent peritoneal dialysis and up to 51.5% (17/33) underwent subsequent renal transplantation (25). On the one hand, due to the very low frequency of renal disease resulting from *NPHS1* variants, individuals and general clinicians are unfamiliar with the disorder as well as its prognosis. On the other hand, most physicians exhibit inadequate recognition and understanding of this illness and its multidisciplinary requirements. We, therefore, need to develop a greater understanding of CNS, both in medical care and patient care personnel, across the nation in the future.

Some potential limitations should be recognized in the current study. First, it was a retrospective analysis where the interventions could not be well controlled. Such as some patients underwent genetic testing by the clinical panel (targeted gene sequencing) rather than all by WES. Although the clinical panel used in our study included 249 genes which almost covered the main responsible genes in hereditary kidney disease, WES is recommended for these patients, if available. Second, it may be underpowered to perform the statistical analyses in comparison between CNS group and non-CNS group due to the overall sample size of the cohort. Significant differences could be made if a larger cohort size is available. Finally, it was a limited number involving only 30 patients collected from 2014 to 2020, although it represents the largest cohort with *NPHS1* variants in China currently. In the next step, it is necessary to expand the cohort size nationwide and longer follow-up is needed.

In conclusion, variants of *NPHS1* not only cause CNS but also NS in early childhood-onset disease. *NPHS1* genetic testing for CNS with onset within 3 months after birth, and also NS with steroid resistance, is helpful for early diagnosis and prognosis evaluation. *NPHS1* variants in Chinese individuals with NS were mainly compound heterozygous variants, and c.928G>A(p.Asp310Asn) in exon 8 may act as a recurrent variant in the Chinese population, followed by c.616C>A(p.Pro206Thr) in exon 6. Steroids and immunosuppressants may have a beneficial effect on selected patients. Kidney transplantation in children with *NPHS1* variants is effective. Efforts are also needed to raise awareness of CNS among patient family members and to improve treatment coverage in China.

## Data Availability Statement

The data that support the findings of this study are available from the corresponding authors upon reasonable request. The datasets presented in this article are not readily available for public repository due to the regulation on the management of human genetic resources from the State Council, CHINA. Requests to access the datasets should be directed to the database for Chinese children renal disease which is publicly available datasets in Chinese language (https://www.ccgkdd.com.cn/).

## Ethics Statement

The studies involving human participants were reviewed and approved by the Institutional Review Board (IRB) of the Children's Hospital of Fudan University (Shanghai, China) (IRB No. 2018286). Written informed consent to participate in this study was provided by the participants' legal guardian/next of kin.

## Author Contributions

HX and XJ designed the study, reviewed, and revised the manuscript. LR, LC, JR, and QS performed the search, performed the analysis and wrote the manuscript, and they have contributed equally to this work. JM, CF, XW, XK, WH, QM, XL, CL, RF, XG, GD, HY, ZH, MH, QL, QZ, and YL collected the data. GL and JL rechecked the data. All authors contributed to the article and approved the submitted version.

## Funding

This work was supported by the Science and Technology Planning Project of Guangzhou, China (Grant No. 202103000001).

## Conflict of Interest

The authors declare that the research was conducted in the absence of any commercial or financial relationships that could be construed as a potential conflict of interest.

## Publisher's Note

All claims expressed in this article are solely those of the authors and do not necessarily represent those of their affiliated organizations, or those of the publisher, the editors and the reviewers. Any product that may be evaluated in this article, or claim that may be made by its manufacturer, is not guaranteed or endorsed by the publisher.
