# Supplementary material for: Genetic Variations and Clinical Features of NPHS1-Related Nephrotic Syndrome in Chinese Children: A Multicenter, Retrospective Study
Source: Front Med (Lausanne). 2021 Nov 11;8:771227. doi: 10.3389/fmed.2021.771227 (PMC8632042; doi:10.3389/fmed.2021.771227)
Supplement: Supplementary file 1 [file Table_1.docx]

**Table S1** 249 genes included in the clinical panel (targeted genes sequencing) in the study

| ACE | BBS9 | COQ2 | FXYD2 | LAMB3 | OCRL | SLC1A1 | UMOD |
| --- | --- | --- | --- | --- | --- | --- | --- |
| ACTN4 | BMP4 | COQ6 | GATA3 | LAMC2 | OFD1 | SLC22A12 | UPK3A |
| ADAMTS13 | BMP7 | COQ9 | GDNF | LMBRD1 | PAX2 | SLC2A2 | VDR |
| AGT | BSND | COQ8A | GLA | LMX1B | PDE6D | SLC2A9 | WAS |
| AGTR1 | C1QA | COQ8B | GLIS2 | LRIG2 | PDGFRA | SLC34A1 | WDPCP |
| AGXT | C1QB | CRB2 | GPC3 | LYZ | PDSS1 | SLC34A3 | WDR19 |
| AHI1 | C1QC | CSPP1 | GRHPR | LZTFL1 | PDSS2 | SLC35A2 | WDR34 |
| ALG1 | C3 | CTBP1 | GRIP1 | MAPK11 | PEX1 | SLC36A2 | WDR35 |
| ALMS1 | CA2 | CTNS | HNF1B | MEFV | PHEX | SLC3A1 | WDR60 |
| AMH | CASR | CUBN | HOGA1 | MKKS | PKD1 | SLC4A1 | WDR73 |
| ANKS6 | CBS | CUL3 | HPSE2 | MKS1 | PKD2 | SLC4A4 | WNK1 |
| ANLN | CC2D2A | CYP27B1 | IFT122 | MMAA | PKHD1 | SLC5A2 | WNK4 |
| APOA1 | CD151 | CYP2R1 | IFT140 | MMAB | PLA2R1 | SLC7A7 | WNT4 |
| APOE | CD2AP | DGKE | IFT172 | MMACHC | PLCE1 | SLC7A9 | WT1 |
| APOL1 | CD46 | DIS3L2 | IFT43 | MMADHC | PMM2 | SMARCAL1 | XDH |
| APRT | CDC5L | DMP1 | IFT80 | MME | PTPRO | SOX17 | XPO5 |
| AQP2 | CEP164 | DSTYK | INF2 | MTHFR | REN | TCTN2 | ZMPSTE24 |
| ARHGAP24 | CEP290 | DYNC2H1 | INPP5E | MTRR | RET | TCTN3 | ZNF423 |
| ARHGDIA | CEP41 | EGF | INVS | MUC1 | ROBO2 | THBD |  |
| ARL6 | CFB | EMP2 | IQCB1 | MUT | RPGRIP1L | THSD7A |  |
| ATP6V0A4 | CFH | ENPP1 | ITGA3 | MYH9 | SALL1 | TMEM138 |  |
| ATP6V1B1 | CFHR5 | EYA1 | ITGA8 | MYO1E | SARS2 | TMEM216 |  |
| AVPR2 | CFI | FAT1 | ITGB4 | NEIL1 | SCARB2 | TMEM231 |  |
| B2M | CHD1L | FGA | KAL1 | NEK1 | SCNN1A | TMEM237 |  |
| B9D1 | CLCN5 | FGF12 | KANK1 | NEK8 | SCNN1B | TMEM67 |  |
| B9D2 | CLCNKA | FGF18 | KANK2 | NPHP1 | SCNN1G | TNXB |  |
| BBS1 | CLCNKB | FGF20 | KANK4 | NPHP3 | SDCCAG8 | TRAP1 |  |
| BBS10 | CLDN16 | FGF23 | KCNA1 | NPHP4 | SIX1 | TRIM32 |  |
| BBS12 | CLDN19 | FGFR1 | KCNJ1 | NPHS1 | SIX2 | TRPC6 |  |
| BBS2 | CNNM2 | FLG | KCNJ10 | NPHS2 | SIX5 | TRPM6 |  |
| BBS4 | COL4A3 | FN1 | KLHL3 | NUP107 | SLC12A1 | TSC2 |  |
| BBS5 | COL4A4 | FRAS1 | LAMA3 | NUP205 | SLC12A3 | TTC21B |  |
| BBS7 | COL4A5 | FREM | LAMB2A3 | NUP93 | SLC17A53 | TTC8 |  |
