# Supplementary material for: Genetic Variations and Clinical Features of NPHS1-Related Nephrotic Syndrome in Chinese Children: A Multicenter, Retrospective Study
Source: Front Med (Lausanne). 2021 Nov 11;8:771227. doi: 10.3389/fmed.2021.771227 (PMC8632042; doi:10.3389/fmed.2021.771227)
Supplement: Supplementary file 2 [file Table_2.docx]

Supplementary Table S2. The information of *NPHS1* variants and in silico predictions (NM_004646.3)

| **Case** | **NHSP1 Mutation** | | | **Mutation Type** | **ACMG classification** | **ACMG Evidence** | **SIFT** | **PolyPhen-2** | **Provean** | **Mutation-Taster2** |
| --- | --- | --- | --- | --- | --- | --- | --- | --- | --- | --- |
|  | **Nucleotide Change** | **Aminoacid Change** | **Location** |  |  |  |  |  |  |  |
| 1 | **IVS25-2T>A*** | / | intron 25 | splice site | VUS | PM2+PM6+PP4 | NA | NA | NA | NA |
|  | c.928C>T | p.Asp310Asn | exon 8 | missense | LP | PM1+PM2 + PP2+PP3+PP4 | D | D | D | D |
| 2 | c.3325C>T | p.Arg1109Ter | exon 26 | nonsense | P | PVS1+PM2+PP5 | NA | NA | NA | A |
|  | **c.3312-2A>T*** | / | / | / | VUS | PM2+PM3+PP4 | NA | NA | NA | D |
| 3 | **c.2590C>T*** | p.Arg864Cys | exon 19 | missense | LP | PM1+PM2+PP2+PP3+PP4 | D | D | D | D |
|  | **c.867G>T*** | p.Trp289Cys | exon 8 | missense | LP | PM2+PM3+PP2+PP3+PP4 | D | D | D | D |
| 4 | c.1394G＞A | p.Cys465Tyr | exon 11 | missense | P | PS1+PM2+PM3+ PP2+PP3+PP4 | D | D | D | D |
|  | c.928G＞A | p.Asp310Asn | exon 8 | missense | P | PS4+PM2 +PM3+ PP2+PP3+PP4 | D | D | D | D |
| 5 | **c.394G>A*** | p.Glu117Lys | exon 3 | missense | VUS | PM1+PM2+PP3+BP4 | D | D | D | D |
|  | **c.1439A>G*** | P.Lys480Thr | exon 11 | missense | VUS | PM2 +PP3+PP4 | D | D | D | D |
|  | **c.1500_1507del*** | p.Gly500fs | exon 12 | frameshift | P | PVS1+ PM2+PP4 | NA | NA | NA | NA |
| 6 | c.3478C>T | p.Arg1160Ter | exon 27 | nonsense | LP | PVS1+PM2 | NA | NA | NA | A |
| 7 | **c.2629-c.2630delA AinsT*** | p.Lys877Xfs*1 | exon 19 | frameshift | P | PVS1+PM2+PM3+PP4 | NA | NA | NA | NA |
|  | c.1315+1G＞A(-) | / | / | / | P | PVS1+PM2+PM3+PP4 | NA | NA | NA | D |
| 8 | **c.2205_2206insTGGAC*** | p.Val736Trpfs*18 | exon 16 | frameshift | P | PVS1+PM2+PM3+PP4 | NA | NA | NA | NA |
|  | c.3478C>T | p.Arg1160Ter | exon 27 | nonsense | LP | PVS1+PM2 | NA | NA | NA | A |
| 9 | c.3213delG | p.Leu1072Phe fs*71 | exon 24 | frameshift | LP | PVS1+PM2 | NA | NA | NA | NA |
|  | c.2663G >A | p.Arg888Thr | exon 19 | missense | P | PS1+PM2+PM3+ PP3+PP4 | T | D | D | D |
| 10 | c.928G＞A | p.Asp310Asn | exon 8 | missense | P | PS4+PM2 +PM3+ PP2+PP3+PP4 | D | D | D | D |
|  | **c.360del C*** | p.Pro120fs | exon 8 | frameshift | P | PVS1+PM2+PM3+PP4 | NA | NA | NA | NA |
|  | c.1240.A＞G | p.Thr414Ala | exon 8 | missense | VUS | PM2+PM3 | T | B | N | N |
| 11 | c.616C>A | p.Pro206Thr | exon 6 | missense | LP | PM1+PM2+PP3+PP4 | D | D | D | D |
|  | **IVS12-10C>A*** | / | intron 12 | splice site | VUS | PM2+PP4 | NA | NA | NA | NA |
| 12 | c.928G＞A | p.Asp310Asn | exon 8 | missense | P | PS4+PM2 +PM3+ PP2+PP3+PP4 | D | D | D | D |
|  | c.2207T>C | p.Val736Ala | exon 16 | missense | P | PM3_strong+PM2+PM1+PM5+PP3 | D | P | D | D |
|  | c.3312-23C>T | / | intron 25 | missense | VUS | PM2+PP4 | NA | NA | NA | NA |
| 13 | **c.2928-2A>C(IVS21) *** | / | intron 21 | splice site | LP | PM2+PM3+PP4+PP5 | NA | NA | NA | D |
|  | c.928G＞A | p.Asp310Asn | exon 8 | missense | P | PS4+PM2 +PM3+ PP2+PP3+PP4 | D | D | D | D |
| 14 | c.802C>T | p.Arg268X | exon 7 | nonsense | LP | PSV1+PM2 | NA | NA | NA | A |
|  | **c.1528T>C*** | p.Ser510Pro | exon 12 | missense | VUS | PM2+PP3 | D | D | D | N |
| 15 | c.2788C>T | p.Gln930X | exon 20 | nonsense | P | PSV1+PM2+PM3 | NA | NA | NA | A |
|  | c.3442delC | p.Gln1148fs | exon 27 | frameshift deletion | P | PSV1+PM2+PM3 | NA | NA | NA | NA |
| 16 | c.2207T>C | p.Val736Ala | exon 16 | missense | P | PM3_strong+PM2+PM1+PM5+PP3 | D | P | D | D |
|  | **c.2210A>C*** | p.His737Pro | exon 16 | missense | VUS | PM2+PM3 | D | B | D | N |
| 17 | c.2212+2_2212+3delTG | NA | NA | splicing | P | PSV1+PM2+PP4 | NA | NA | NA | NA |
|  | **c.1409G>A*** | p.Gly470Asp | exon 11 | missense | VUS | PM2+PM3 | D | D | D | D |
| 18 | c.1440+1G>A | NA | intron 11 | splicing | P | PSV1+PM2+PM3 | NA | NA | NA | D |
|  | c.928G>A | p.Asp310Asn | exon 8 | missense | P | PS4+PM2 +PM3+ PP2+PP3+PP4 | D | D | D | D |
| 19 | **c.741G>A*** | p.Trp247X | exon 7 | nonsense | P | PVS1+PM2+PM3 | NA | NA | NA | A |
|  | c.928G>A | p.Asp310Asn | exon 8 | missense | P | PS4+PM2 +PM3+ PP2+PP3+PP4 | D | D | D | D |
| 20 | **c.3144delG*** | p.Gln1048fs | exon 23 | frameshift deletion | P | PVS1+PM2+PM3 | NA | NA | NA | NA |
|  | **c.514delA*** | p.Thr172fs | exon 4 | frameshift deletion | P | PVS1+PM2+PM3 | NA | NA | NA | NA |
| 21 | **c.1699T>C*** | p.Cys567Arg | exon 13 | missense | VUS | PM1+PM2+PP3 | D | D | D | D |
|  | c.3523_3524del | p.Leu1175ValfsTer2 | exon 28 | frameshift deletion | LP | PVS1+PM2 | NA | NA | NA | NA |
| 22 | **c. 1531C ＞ T*** | p.Arg511X,731 | exon 12 | frameshift deletion | LP | PVS1+PM2 | NA | NA | NA | A |
|  | **c.2071+2T>C*** | NA | NA | splicing | P | PVS1+PM2+PP4+PP5+PP3 | NA | NA | NA | D |
| 23 | c.2783C>A | p.Ser928X | exon 20 | nonsense | P | PVS1+PM2+PM3 | NA | NA | NA | A |
|  | c.928G>A | p.Asp310Asn | exon 8 | missense | P | PS4+PM2 +PM3+ PP2+PP3+PP4 | D | D | D | D |
| 24 | c.1219C>T | p. Arg407Trp | exon 10 | missense | P | PS1+PM1+PM2+PM3+PP3 | D | D | D | D |
|  | **dup(exon23-28)*** | NA | exon 23-28 | duplication | LP | PVS1-Strong+PM2 | NA | NA | NA | NA |
| 25 | c.616C＞A | p.Pro206Thr | exon 6 | missense | LP | PM1+PM2+PP3+PP4 | D | D | D | D |
|  | **c.472G>T*** | p.Val158Fhe | exon 4 | missense | VUS | PM1+PM2 | D | D | D | D |
| 26 | c.2207T>C | p.Val736Ala | exon 16 | missense | P | PM3_strong+PM2+PM1+PM5+PP3 | D | P | D | D |
|  | c.616C>A | p.Pro206Thr | exon 6 | missense | LP | PM1+PM2+PP3+PP4 | D | D | D | D |
| 27 | c.2783C>A | p.Ser928X | exon 20 | nonsense | P | PVS1+PM2+PM3 | NA | NA | NA | A |
|  | c.139delG | p.Ala47ProfsTer81 | exon 2 | frameshift | LP | PVS1+PM2 | NA | NA | NA | NA |
| 28 | c.3250dupG | p.Val1084fs | exon 24 | frameshift insertion | LP | PVS1+PM2 | NA | NA | NA | NA |
|  | **c.2380T>C*** | p.Ser794Pro | exon 18 | missense | VUS | PM1+PM2+PP3 | D | D | N | N |
| 29 | c.1394G＞A | p.Cys465Tyr | exon 11 | missense | P | PS1+PM2+PM3+ PP2+PP3 | D | D | D | D |
|  | c.616C＞A | p.Pro206Thr | exon 6 | missense | LP | PM1+PM2+PP3+PP4 | D | D | D | D |
| 30 | **c.3110_3166del*** | / | exon 23 | nonframeshift deletion | P | PSV1+PM2+PM4 | NA | NA | NA | NA |

*Novel variants in bold

**ACMG classification**: P: Pathogenic; LP: Likely Pathogenic; VUS: Variants of uncertain significance; NA: Not available; D: Damaging; T: Tolerated; P in **PolyPhen-2**: possibly damaging; B: Benign; A: disease causing automatic; N in Mutation-Taster2: polymorphism; N in Provean: Neutral
